# Brain Tissue Conductivity Measurements with MR-Electrical Properties Tomography: An In Vivo Study

**DOI:** 10.1007/s10548-020-00813-1

**Published:** 2020-12-08

**Authors:** Stefano Mandija, Petar I. Petrov, Jord J. T. Vink, Sebastian F. W. Neggers, Cornelis A. T. van den Berg

**Affiliations:** 1 Computational Imaging Group for MR Diagnostic & Therapy, Center for Image Sciences, University Medical Center Utrecht, Heidelberglaan 100, Utrecht, 3584 CX The Netherlands; 2 Division of Imaging & Oncology, Department of Radiotherapy, University Medical Center Utrecht, Heidelberglaan 100, Utrecht, 3584 CX The Netherlands; 3 Rudolf Magnus Institute of Neuroscience, University Medical Center Utrecht, Heidelberglaan 100, Utrecht, 3584 CX The Netherlands

**Keywords:** Conductivity, Electrical properties, MR-EPT, Brain

## Abstract

First in vivo brain conductivity reconstructions using Helmholtz MR-Electrical Properties Tomography (MR-EPT) have been published. However, a large variation in the reconstructed conductivity values is reported and these values differ from ex vivo conductivity measurements. Given this lack of agreement, we performed an in vivo study on eight healthy subjects to provide reference in vivo brain conductivity values. MR-EPT reconstructions were performed at 3 T for eight healthy subjects. Mean conductivity and standard deviation values in the white matter, gray matter and cerebrospinal fluid (σ_WM_, σ_GM_, and σ_CSF_) were computed for each subject before and after erosion of regions at tissue boundaries, which are affected by typical MR-EPT reconstruction errors. The obtained values were compared to the reported ex vivo literature values. To benchmark the accuracy of in vivo conductivity reconstructions, the same pipeline was applied to simulated data, which allow knowledge of ground truth conductivity. Provided sufficient boundary erosion, the in vivo σ_WM_ and σ_GM_ values obtained in this study agree for the first time with literature values measured ex vivo. This could not be verified for the CSF due to its limited spatial extension. Conductivity reconstructions from simulated data verified conductivity reconstructions from in vivo data and demonstrated the importance of discarding voxels at tissue boundaries. The presented σ_WM_ and σ_GM_ values can therefore be used for comparison in future studies employing different MR-EPT techniques.

**Supplementary information:**

The online version of this article (10.1007/s10548-020-00813-1) contains supplementary material, which is available to authorized users.

## Introduction

Tissue Electrical Properties (EPs: conductivity and permittivity) regulate how electromagnetic fields, such as the MR radiofrequency fields (RF: 64–300 MHz) (Katscher et al. 2009; Voigt et al. 2011), interact with the human body. Dielectric probe measurements have shown a significant change of these properties as a function of frequency (Gabriel et al. 1996a, b, c). For medical applications, there are two major frequency ranges that are of interest: low-frequencies (LF: up to kHz) and high-frequencies (radiofrequencies, RF: hundreds MHz).

Low-frequency tissue conductivity measurements have been proven to be feasible with Electrical Impedance Tomography (EIT) and MR-EIT (Metherall et al. 1996; Ider and Onart 2004; Seo et al. 2005; Gao et al. 2005; Woo and Seo 2008). More recently, to avoid direct current injection in the body required in EIT, it has been proposed to inductively induce currents by exploiting the MRI gradient system or with Transcranial Magnetic Stimulation devices (Mandija et al. 2014, 2015a, 2016b; Gibbs and Liu 2015a). Although this is an appealing idea as additional hardware for direct current injection is not needed, these MR-based methods lack sufficient sensitivity (Gibbs and Liu 2015b; Mandija et al. 2015b, 2016a; Oran and Ider 2016).

While non-invasive LF conductivity measurements are not feasible using only MRI systems, in the last decade it has been shown that non-invasive MR-based RF conductivity measurements are feasible (Wen 2003; Voigt et al. 2009). This technique is known as MR-Electrical Properties Tomography (MR-EPT). Knowledge of subject-specific RF conductivity is important to correctly assess the local specific absorption rate (SAR) for RF safety (Katscher et al. 2009; Murbach et al. 2011; Zhang et al. 2013b). Furthermore, it has been shown that at RF frequencies tumors have different conductivity values than normal tissues (Schepps and Foster 1980; Surowiec et al. 1988). Hence, in vivo RF conductivity measurements could be used as a biomarker for diagnostic purposes (Surowiec et al. 1988; van Lier et al. 2011; Katscher et al. 2012, 2015; Shin et al. 2015).

MR-EPT aims to reconstruct tissue EPs at RF frequencies from non-invasive MR measurements of complex ${\text{B}}_{1}^{+}$ fields using clinical MRI coils (Katscher et al. 2013; Katscher and van den Berg 2017). Standard MR-EPT reconstruction methods are based on the Helmholtz equation (Katscher et al. 2013; Katscher and van den Berg 2017). All these Helmholtz-based methods require the computation of spatial derivatives on measured data. In particular, as it appears in the Helmholtz equations, the computation of the second order spatial derivative of the complex ${\text{B}}_{1}^{+}$ field is highly sensitive to noise in the MR measurements (Shin et al. 2014; Lee et al. 2015; Mandija et al. 2018). To reduce the impact of noise, large derivative kernels and image filters are often adopted at the cost of numerical errors at boundaries, where spatial extension increases with increasing kernel/filter size (Seo et al. 2012; Duan et al. 2016; Gurler and Ider 2016). However, this limits the accuracy of MR-EPT reconstructions on a voxel basis.

Nevertheless, Helmholtz MR-EPT allows inference of the mean conductivity values for homogeneous regions that are larger than the spatial extent of the finite difference kernel (Shin et al. 2015). This information can be used to assess mean in vivo tissue conductivity values and verify the reported literature values. This is relevant as literature values used as a reference in MR-EPT studies pertain to excised tissues (Gabriel et al. 1996a, b, c), for which EPs properties might differ from in vivo tissues.

Yet, as highlighted in three recently published works (Katscher and van den Berg 2017; Hancu et al. 2018; McCann et al. 2019), the number of studies showing in vivo RF conductivity reconstructions is limited, while permittivity reconstructions are not feasible. In particular, for brain tissues, the number of test subjects reported in these studies is very small (Voigt et al. 2011; Zhang et al. 2013a; Michel et al. 2016; Tha et al. 2018), and in vivo studies on groups of healthy subjects studies are currently missing. In addition to the scarce amount of in vivo brain conductivity reconstructions, the results presented in these studies lack agreement (McCann et al. 2019). A large variation in the reconstructed conductivity values is reported and these results substantially differ from ex vivo values. We hypothesize that one cause of the reported variation in conductivity values is the way regions at tissue boundaries are handled by different MR-EPT reconstruction pipelines. These regions are affected by well-known MR-EPT reconstruction errors, which alter the calculation of mean conductivity values if they are not handled correctly. Thus, although highly desired, knowledge on in vivo tissue RF conductivity values is limited. For this reason, ex vivo literature values are used as a reference for various in vivo applications, such as RF safety assessment (Murbach et al. 2011; Neufeld et al. 2011; Homann et al. 2011).

Given this lack of agreement, we performed an in vivo study to provide reference brain RF conductivity values of the white and gray matter (σ_WM_, σ_GM_). Helmholtz-based conductivity reconstructions on eight healthy subjects are presented and the reconstructed mean σ_WM_ and σ_GM_ values are compared to literature. To investigate the impact of boundary errors on mean conductivity values, mean σ_WM_ and σ_GM_ values are computed with and without exclusion of regions affected by boundary errors. To validate the accuracy of in vivo conductivity reconstructions, an electromagnetic simulation study was also performed. To the best of our knowledge, this is the first study performing conductivity reconstructions in the brain for a group of healthy subjects.

## Methods

Following ethical protocols approved by the local IRB of the UMC Utrecht, MRI measurements were performed on eight volunteers (2 male, 6 female, mean age 21.7, standard deviation 2.3) using a clinical 3 T MR-scanner (Achieva, Philips, Best, The Netherlands) and an 8-channel receive head coil (the birdcage coil was used for transmission in quadrature mode). To correct for non-uniform receiver coil profiles, and to convert the receive phase measured with the head coil to the body coil, as if the body coil would have been used both for transmitting and receiving, the vendor specific algorithm CLEAR (Constant Level of Appearance) was automatically run at the scanner. To minimize head motion during the MRI exam, the head of the subjects was fixated inside the head coil with pads.

The ${\text{B}}_{1}^{+}$ magnitude was measured using a 3D-dual-TR sequence (Yarnykh 2007): TR_1_/TR_2_/TE = 50/250/2.5 ms, flip angle = 65°, field of view (FOV) = 240 × 240 × 90 mm^3^, voxel size = 2.5 × 2.5 × 3 mm^3^, about 14 min scan time.

The ${\text{B}}_{1}^{+}$ phase was approximated with half of the transceive phase (Mandija et al. 2018). To map the transceive phase, two phase maps acquired using two 2D-single-echo Spin-Echo sequences with opposite readout gradient polarities were combined ($\varphi ^{ \pm }  = \frac{{(\varphi _{{{\text{spin\_echo}}\_1}}  + \varphi _{{{\text{spin\_echo}}\_2}} )}}{2}$), thus minimizing the impact of eddy-currents related artifacts (Mandija et al. 2015b). The adopted sequence parameters were: TR/TE = 800/6 ms, FOV = 240 × 240 × 90 mm^3^, voxel size = 2.5 × 2.5 × 2.5 mm^3^, slice gap = 0.5 mm, number-of-signal averaging (NSA) = 2, about 5 min scan time for each Spin-Echo sequence.

In vivo conductivity reconstructions were performed according to:

1 $$ \sigma \left( r \right) = \frac{1}{{\mu _{0} \omega }}Im\left( {\frac{{\nabla ^{2} B_{1}^{ + } (r)}}{{B_{1}^{ + } (r)}}} \right) $$

with $\omega$: Larmor angular frequency, ${\mu }_{0}$: free space permeability, and **r**: x,y,z-coordinates. Second order spatial derivatives were computed using a noise-robust, in-plane derivative kernel (K_Large_: 7 × 7 voxels) (Mandija et al. 2018). A 3D derivative kernel could not be used since the MR sequences used to compute the transceive phase ($\varphi ^{ \pm }$) demonstrated well-known random phase offsets between slices. This prevented computation of spatial derivatives through slices. Gibbs ringing correction and k-space Gaussian apodization were performed to minimize the impact of high frequency spatial fluctuations in conductivity reconstructions (Mandija et al. 2018).

First, mean and standard deviation of the reconstructed σ_WM_, σ_GM_, and σ_CSF_ were computed for the WM, GM and CSF of each subject and among subjects. For this purpose, tissue segmentation was performed for each subject in SPM12 (WTCN, UCL, London, UK) using the Spin-Echo volumes acquired to reconstruct $\varphi ^{\pm }$. Only the voxels with a probability value (P) > 99% to belong to a certain tissue were considered, thus avoiding voxels at interfaces affected by partial volume.

Then, mean and standard deviation of σ_WM_, σ_GM_ were recomputed after additional erosion of the WM and GM masks previously obtained from SPM12 in order to avoid regions at tissue boundaries that are affected by typical MR-EPT boundary errors. In particular, for each subject, each slice of the previously computed WM and GM masks was independently eroded in MatlabMatlab R2019a, The MathWorks Inc) using the predefined Matlab function imerode (ErodedMask = imerode(OriginaMask, structuring element), with structuring element = strel (disk, 2)) (see supplementary material, parts 1 to 3).

The obtained mean σ_WM_ and σ_GM_ values were therefore compared to the reported ex vivo literature values. Unfortunately, this characterization could not be done for the CSF due its limited spatial extension and well known MRI acquisition artifacts (Katscher et al. 2018).

**Fig. 1:**
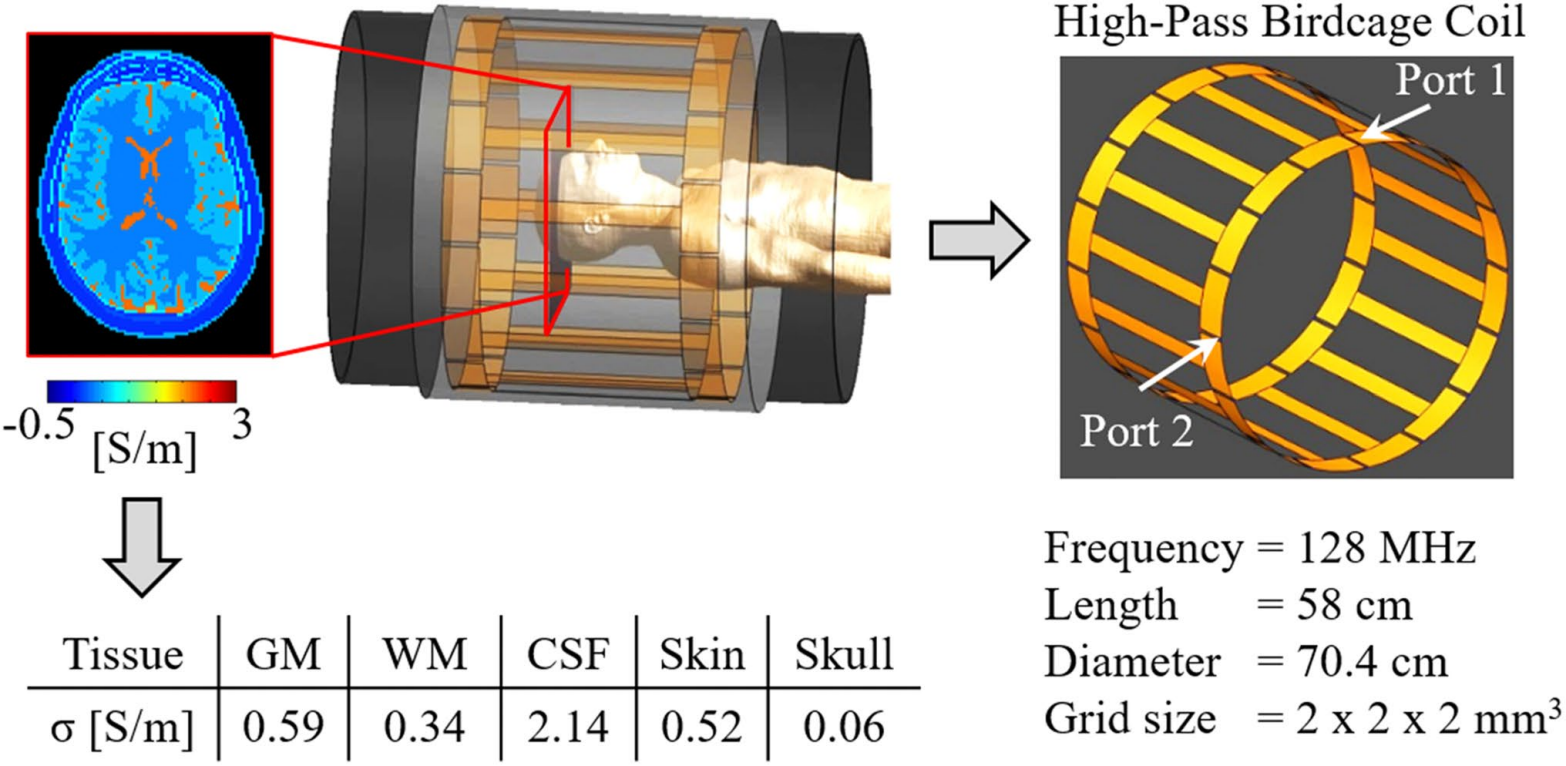
Simulation setup used for the FDTD simulation on the Duke head model and ground truth conductivity values at 128 MHz

To benchmark the accuracy of the in vivo conductivity reconstructions, the same pipeline used for the MRI data was applied to FDTD simulated complex ${\text{B}}_{1}^{+}$ data in Sim4Life (ZMT AG, Zurich, Switzerland) (same in-plane derivative kernel and spatial erosion), as simulated data allow knowledge of the ground truth conductivity. For these sophisticated electromagnetic simulations, the Duke model was used (Christ et al. 2010), while the simulated transmit coil setup was similar to the one used for the MRI measurements (see Fig. 1). Gaussian noise was added to the real and imaginary parts of the simulated complex ${\text{B}}_{1}^{+}$ data (SNR = 50), thus mimicking clinical SNR levels achievable for in vivo EPT measurements (Mandija et al. 2018). The mean σ_WM_ and σ_GM_ were computed over the whole head model after the same in-plane erosion used for the in vivo reconstructions was ultimately applied. Additionally, in the supplementary material (parts 1 and 2) we have characterized the impact of using the in-plane derivative kernel K_Large_ instead of a 3D K_Large_ derivative kernel.

## Results

**Fig. 2:**
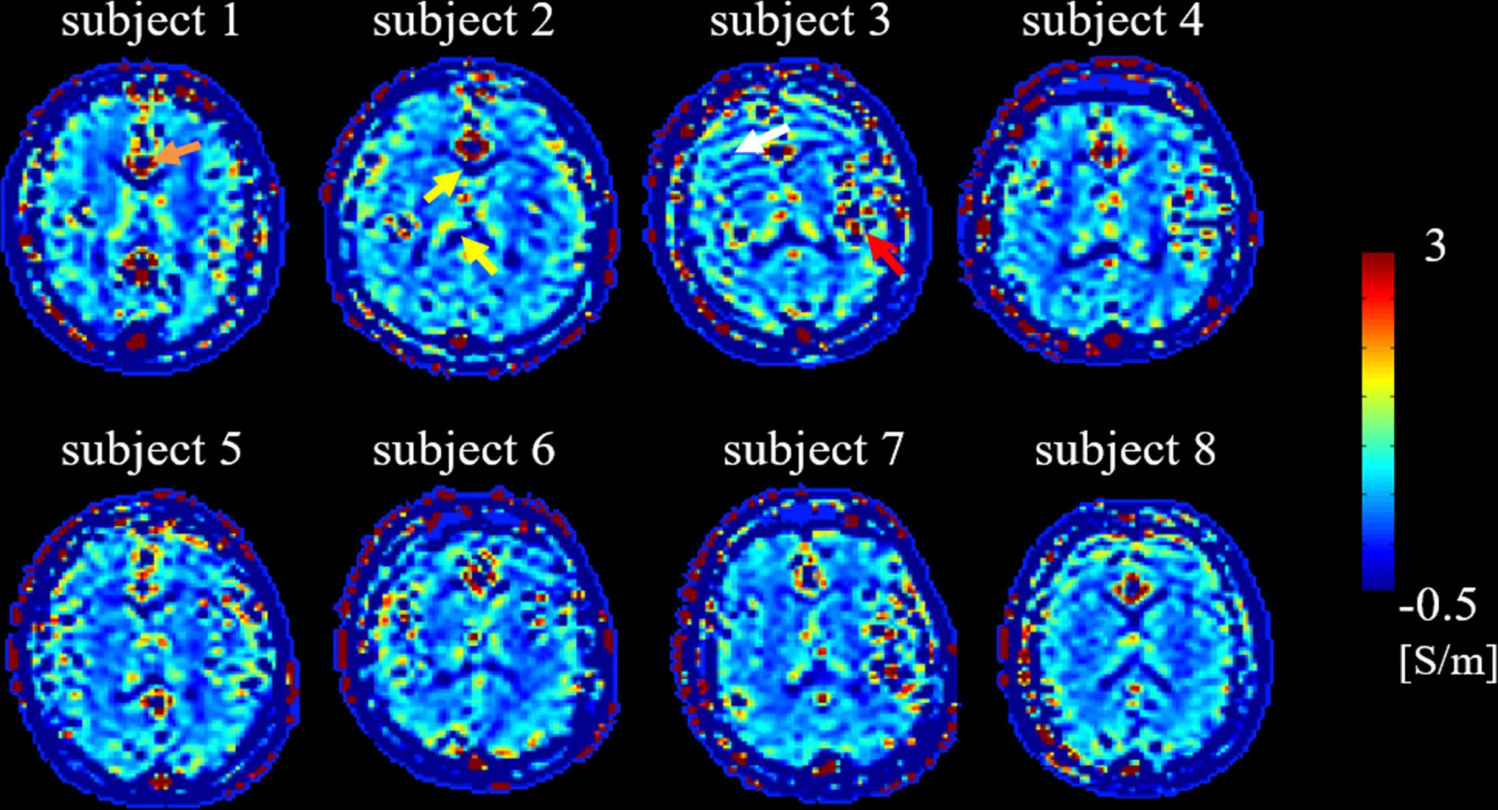
For each subject, one slice of the in vivo conductivity reconstructions is shown as example

In Fig. 2, a conductivity map (transversal view) is shown for each volunteer as example for visual inspection. These maps are shown on brain slices taken at the level of the ventricles, and show comparable reconstruction quality among volunteers. Boundary errors are noticeable around the ventricles (e.g. subject 2, yellow arrows) and on the lateral sides at the interface between CSF/GM/WM (e.g. subject 3, red arrow). Blood pulsation related artifacts are also visible around major vessels (e.g. subject 1, orange arrow). The usage of pads to fixate the head of the volunteers was successful in all volunteers, except for subject 3, where a few slices showed a motion related artifact in the conductivity map (white arrow, negligible impact on the reconstructed mean conductivity values after boundary erosion).

Mean σ_WM_ and σ_GM_ values and standard deviations are reported in Table 1, before and after erosion of regions at tissue boundaries, which are affected by well-known reconstructions errors. This allows assessment of the impact of boundary errors on the computed mean conductivity values.

**Table 1:** Mean conductivity values (S/m) and standard deviations (inside brackets) for each subject and among all subjects without (left side) and with (right side) boundary erosion

Subject	Without boundary erosion			With boundary erosion		
	GM	WM	CSF	GM	WM	CSF
1	0.37 (1.41)	0.43 (0.45)	0.29 (2.76)	0.54 (0.68)	0.32 (0.24)	–
2	0.52 (0.81)	0.38 (0.41)	0.18 (1.98)	0.56 (0.73)	0.31 (0.22)	–
3	0.41 (1.07)	0.38 (0.39)	0.36 (1.93)	0.55 (1.05)	0.29 (0.26)	–
4	0.46 (1.16)	0.36 (0.39)	0.39 (2.11)	0.43 (1.07)	0.29 (0.21)	–
5	0.55 (0.81)	0.41 (0.32)	0.52 (1.69)	0.54 (0.65)	0.31 (0.20)	–
6	0.55 (0.87)	0.39 (0.39)	0.40 (1.92)	0.59 (0.84)	0.32 (0.28)	–
7	0.52 (0.96)	0.40 (0.33)	0.68 (2.13)	0.55 (0.79)	0.31 (0.18)	–
8	0.46 (1.00)	0.42 (0.42)	0.22 (1.64)	0.53 (1.23)	0.32 (0.26)	–
Mean	0.48 (1.03)	0.40 (0.39)	0.38 (2.05)	0.53 (0.90)	0.31 (0.23)	–
Literature (ex vivo)^a^	0.59	0.34	2.14	0.59	0.34	2.14

^a^Literature (ex vivo) values were taken from (Gabriel et al. 1996a, b, c)

Mean σ_WM_, σ_GM_, and σ_CSF_ values and standard deviations (without boundary erosion) are reported in Table 1, left-side. Mean σ_WM_ and σ_GM_ values show respectively ~ 30% over/underestimation compared to the reported literature value, while mean σ_CSF_ values are instead highly underestimated compared to literature values due to severe boundary errors.

In Table 1, right side, mean σ_WM_ and σ_GM_ values and standard deviations are reported after eroding the WM and GM masks to exclude regions affected by boundary errors. The reported mean and standard deviation values averaged over the eight subjects are 0.31 ± 0.23 S/m and 0.53 ± 0.90 S/m for the WM and GM, respectively. Additionally, in the supplementary material (part 4) mean and standard deviation values are computed for each subject in different regions of interest taken on the slices shown in Fig. 2, and, in supplementary material (part 5), mean and standard deviation values are also computed for different regions of interest taken on different slices throughout the brain of subject 4 as example.

**Fig. 3:**
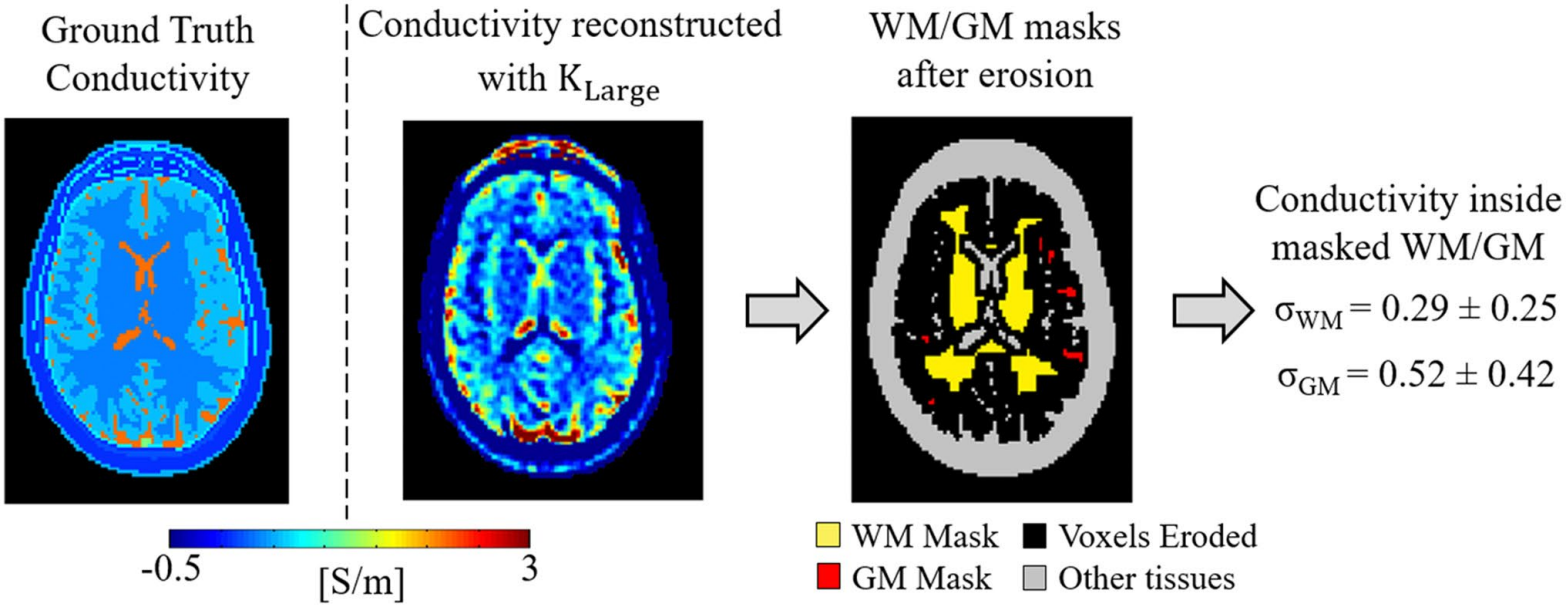
One example slice of conductivity reconstructions for the Duke simulations. Mean WM and GM conductivity values and standard deviations (inside brackets) are also reported, after the same erosion applied for the in vivo conductivity reconstructions was performed

The results from simulations performed to benchmark the accuracy of the in vivo reconstruction pipeline are reported in Fig. 3, where the reconstructed conductivity is shown for one slice together with the mean σ_WM_ and σ_GM_ values of the whole Duke head computed after the same erosion applied for the in vivo conductivity reconstructions was performed. The obtained mean σ_WM_ and σ_GM_ values agree with the reconstructed values in vivo. These values show, however, a small underestimation (~ 10%) with respect to the input ground truth conductivity values. This is known to be caused by the fact that conductivity contribution arising from derivatives through slices are neglected (supplementary material part 2), as these derivatives cannot be computed for the in vivo case. Ultimately, this explains the small underestimation in the reconstructed in vivo σ_WM_ and σ_GM_ values with respect to literature values measured ex vivo.

## Discussion

The presented study aims at providing reference conductivity values for the brain white and gray matter. In vivo Helmholtz-based MR-EPT reconstructions on eight healthy subjects were performed from MR measurements at 3 T. The reconstructed mean conductivity values are in line with the reported literature conductivity values (measured ex vivo) and can therefore be used for comparison in future studies employing different MR-EPT techniques.

A major source of error in Helmholtz-based MR-EPT reconstructions is the computation of spatial derivatives on measured data. To mitigate the noise amplification cause by this derivative operation, relatively large finite difference kernels such as the adopted K_Large_ or the Savitzky-Golay kernels (Lee et al. 2015; Mandija et al. 2018) are commonly used. This leads inevitably to inaccurate MR-EPT reconstructions on a voxel basis and numerical boundary errors when spatial derivatives are computed for voxels at tissue boundaries (see Fig. 3, ground truth vs reconstructed conductivity maps), as large derivative kernels would also include voxels belonging to different tissues. To avoid the use of derivative kernels, inverse MR-EPT reconstruction approaches have been suggested (Balidemaj et al. 2015; Borsic et al. 2016; Ropella and Noll 2017). However, these inverse models require electromagnetic quantities that are not always accessible with MRI (incident electric field) (Balidemaj et al. 2015). Better quality conductivity reconstructions from simulated data have been recently obtained with deep-learning based approaches (Hampe et al. 2019; Leijsen et al. 2019; Mandija et al. 2019), but their generalization to in vivo cases remains challenging due to the lack of accurate in vivo reconstructions to train neural networks.

Contrary to the studies above, the presented study does not introduce a new methodology for MR-EPT, nor does it aim at solving the noise amplification problem of Helmholtz-based MR-EPT reconstructions. Instead, it focuses on providing reference mean conductivity values of the brain WM/GM tissues using the well-known and widely implemented Helmholtz-based MR-EPT method. As shown in this work, in vivo mean σ_WM_ and σ_GM_ values reconstructed using Helmholtz-based MR-EPT are erroneous if regions at tissue boundaries, which are affected by well-known MR-EPT reconstruction errors, are not excluded. Instead, provided sufficient boundary erosion to avoid these regions, mean σ_WM_ and σ_GM_ values are in good agreement with the reported literature values measured ex vivo. This indicates that: (1) boundary erosion is crucial for correct quantification of mean conductivity values; (2) the way boundaries are handled has severe impact on the reconstructed mean conductivity values. This latter observation might explain the large variation in the reported literature brain conductivity values using MR-EPT, as these studies use different derivative kernels, which have different impact on conductivity reconstructions at tissue boundaries.

Unfortunately, this erosion cannot be applied to the CSF due to its limited spatial extension. Smaller resolutions and small derivative kernels should be adopted to correctly quantify σ_CSF_, but this would lead to conductivity maps highly corrupted by noise.

From the presented in vivo results in Table 1, we can also observe that the standard deviations are comparable/higher than the mean conductivity values before erosions are applied. Instead, after boundary erosions are applied, the standard deviations are reduced, especially for the WM. These reflect the well-known impact of noise amplification in the reconstructed conductivity maps. It has to be noted that here we refrained from applying imaging filters for denoising purposes in post-processing. This explains the higher standard deviations observed in this study compared to other studies, where imaging filters are applied. Yet, if correctly applied, these imaging filters should only lead to lower standard deviations and thus nicer looking images, but should not affect the mean conductivity values, otherwise correct quantification of mean conductivity values would be hampered. For an example of the impact of different denoising filters on EPT reconstructions, we refer to Jung et al. 2020.

Therefore, given the absence of gold standard measurements for in vivo conductivity reconstructions, we believe that the observed agreement between in vivo mean σ_WM_ and σ_GM_ values and ex vivo literature mean conductivity values gives confidence on in vivo mean σ_WM_ and σ_GM_ values for healthy subjects. These values could therefore serve as reference for future studies employing different MR-EPT techniques. Furthermore, they might also give more confidence in adopting literature values to assess RF safety (Neufeld et al. 2011; Homann et al. 2011).

## Conclusions

In this work, we have demonstrated that boundary erosion is crucial in Helmholtz-based MR-EPT to correctly quantify mean conductivity values in the gray and white matter.

If boundaries are not handled correctly, erroneous mean conductivity values are obtained. This can explain the large variability among the brain conductivity values reported in literature.

The in vivo σ_WM_ and σ_GM_ values obtained in this study are in line with the reported literature values measured ex vivo. The accuracy of the reconstruction procedure using a 2D derivative kernel was verified in simulation settings. The presented σ_WM_ and σ_GM_ values provide additional evidences on in-vivo WM GM conductivity values and can be used for comparison in future studies employing different MR-EPT techniques.

## Supplementary information

Below is the link to the electronic supplementary material.

Supplementary material 1 (PDF 1746 kb)

## References

[CR1] Balidemaj E, van den Berg CAT, Trinks J (2015). CSI-EPT: a contrast source inversion approach for improved MRI-based electric properties tomography. IEEE Trans Med Imaging.

[CR2] Borsic A, Perreard I, Mahara A, Halter RJ (2016). An inverse problems approach to MR-EPT image reconstruction. IEEE Trans Med Imaging.

[CR3] Christ A, Kainz W, Hahn EG, Honegger K, Zefferer M, Neufeld E, Rascher W, Janka R, BautzW CJ, Kiefer B, Schmitt P, Hollenbach HP, Shen J, Oberle M, Szczerba D, Kam A, Guag JW, Kuster N (2010). The Virtual Family - Development of surface-based anatomical models of two adults and two children for dosimetric simulations. Phys Med Biol.

[CR4] Duan S, Xu C, Deng G (2016). Quantitative analysis of the reconstruction errors of the currently popular algorithm of magnetic resonance electrical property tomography at the interfaces of adjacent tissues. NMR Biomed.

[CR5] Gabriel C, Gabriel S, Corthout E (1996). The dielectric properties of biological tissues: I. Literature survey. Phys Med Biol.

[CR6] Gabriel S, Lau RW, Gabriel C (1996). The dielectric properties of biological tissues: II. Measurements in the frequency range 10 Hz to 20 GHz. Phys Med Biol.

[CR7] Gabriel S, Lau RW, Gabriel C (1996). The dielectric properties of biological tissues: III. Parametric models for the dielectric spectrum of tissues. Phys Med Biol.

[CR8] Gao N, Zhu S, He B (2005). Estimation of electrical conductivity distribution within the human head from magnetic flux density measurement. Phys Med Biol.

[CR9] Gibbs E, Liu C (2015). Feasibility of imaging tissue electrical conductivity by switching field gradients with MRI. Tomography.

[CR10] Gibbs E, Liu C (2015a) Simulating charge at electrical property interfaces. In: Proceedings of the 23nd science meeting international society for magnetic resonance in medicine Toronto, Canada, p 3290

[CR11] Gurler N, Ider YZ (2016). Gradient-based electrical conductivity imaging using MR phase. Magn Reson Med.

[CR12] Hampe N, Katscher U, van den Berg CAT, et al (2019) Deep learning brain conductivity mapping using a patch -based 3D U- net. arXiv 1908.04118

[CR13] Hancu I, Liu J, Hua Y, Lee S-K (2018). Electrical properties tomography: available contrast and reconstruction capabilities. Magn Reson Med.

[CR14] Homann H, Börnert P, Eggers H (2011). Toward individualized SAR models and in vivo validation. Magn Reson Med.

[CR15] Ider YZ, Onart S (2004). Algebraic reconstruction for 3D magnetic resonance-electrical impedance tomography (MREIT) using one component of magnetic flux density. Physiol Meas.

[CR16] Jung KJ, Mandija S, Kim JH, et al (2020) Improving phase-based conductivity reconstructions by means of deep learning-based denoising of B1+ phase data. In: Proceedings of the 20th science meeting international society for magnetic resonance in medicine, p 181 10.1002/mrm.28826 33949721

[CR17] Katscher U, van den Berg CAT (2017). Electric properties tomography: biochemical, physical and technical background, evaluation and clinical applications. NMR Biomed.

[CR18] Katscher U, Voigt T, Findeklee C (2009). Determination of electric conductivity and local SAR via B1 mapping. IEEE Trans Med Imaging.

[CR19] Katscher U, Djamshidi K, Voigt T, et al (2012) Estimation of breast tumor conductivity using parabolic phase fitting. In: Proceedings of the 20th science meeting international society for magnetic resonance in medicine Melbourne, Victoria, Australia, p 2335

[CR20] Katscher U, Kim D-H, Seo JK (2013). Recent progress and future challenges in MR electric properties tomography. Comput Math Methods Med.

[CR21] Katscher U, Abe H, Ivancevic MK, Keupp J (2015) Investigating breast tumor malignancy with electric conductivity measurements. In: Proceedings of the 23nd science meeting international society for magnetic resonance in medicine Toronto, Canada, p 3306

[CR22] Katscher U, Stehning C, Tha KK (2018) The impact of CSF pulsation on reconstructed brain conductivity. In: Proceedings of the 26th science meeting international society for magnetic resonance in medicine Paris, France, p 546

[CR23] Lee S, Bulumulla S, Hancu I (2015). Theoretical investigation of random noise-limited signal-to-noise ratio in MR-based electrical properties tomography. IEEE Trans Med Imaging.

[CR24] Leijsen RL, Van Den Berg C, Webb AG (2019). Combining deep learning and 3D contrast source inversion in MR-based electrical properties tomography. NMR Biomed.

[CR25] Mandija S, van Lier ALHMW, Thielscher A, et al (2014) Characterizing electrical interactions of tissues with time varying gradient fields: simulations and measurements. In: Proceedings of the 22nd Science meeting international society for magnetic resonance in medicine Milano, Italy, p 639

[CR26] Mandija S, van Lier ALHMW, Katscher U (2015). A geometrical shift results in erroneous appearance of low frequency tissue eddy current induced phase maps. Magn Reson Med.

[CR27] Mandija S, Petrov PI, Neggers SFW, et al (2015a) MR guidance of TMS for a patient specific treatment plan: MR based TMS field measurements and electromagnetic simulations. In: Proceedings of the 23nd Science meeting international society for magnetic resonance in medicine Toronto, Canada, p 931

[CR28] Mandija S, Petrov PI, Neggers SFW (2016). MR-based measurements and simulations of the magnetic field created by a realistic transcranial magnetic stimulation (TMS) coil and stimulator. NMR Biomed.

[CR29] Mandija S, Petrov PI, Neggers SFW (2016). Noninvasive electric current induction for low-frequency tissue conductivity reconstruction: is it feasible with a TMS-MRI setup?. Tomography.

[CR30] Mandija S, Sbrizzi A, Katscher U (2018). Error analysis of helmholtz-based MR-electrical properties tomography. Magn Reson Med.

[CR31] Mandija S, Meliadò EF, Huttinga NRF (2019). Opening a new window on MR-based electrical properties tomography with deep learning. Sci Rep.

[CR32] McCann H, Pisano G, Beltrachini L (2019). Variation in reported human head tissue electrical conductivity values. Brain Topogr.

[CR33] Metherall P, Barber DC, Smallwood RH, Brown BH (1996). Three-dimensional electrical impedance tomography. Nature.

[CR34] Michel E, Hernandez D, Lee SY (2016). Electrical conductivity and permittivity maps of brain tissues derived from water content based on T 1 -weighted acquisition. Magn Reson Med.

[CR35] Murbach M, Cabot E, Neufeld E (2011). Local SAR enhancements in anatomically correct children and adult models as a function of position within 1.5 T MR body coil. Prog Biophys Mol Biol.

[CR36] Neufeld E, Gosselin M-C, Murbach M (2011). Analysis of the local worst-case SAR exposure caused by an MRI multi-transmit body coil in anatomical models of the human body. Phys Med Biol.

[CR37] Oran OF, Ider YZ (2016). Feasibility of conductivity imaging using subject eddy currents induced by switching of MRI gradients. Magn Reson Med.

[CR38] Ropella KM, Noll DC (2017). A regularized, model-based approach to phase-based conductivity mapping using MRI. Magn Reson Med.

[CR39] Schepps JL, Foster KR (1980). The Uhf and microwave dielectric properties of normal and tumor tissues variation in dielectric properties with tissue water content. Phys Med Biol.

[CR40] Seo JK, Kwon O, Woo EJ (2005). Magnetic resonance electrical impedance tomography (MREIT): conductivity and current density imaging. J Phys Conf Ser.

[CR41] Seo JK, Kim M-O, Lee J (2012). Error analysis of nonconstant admittivity for MR-based electric property imaging. IEEE Trans Med Imaging.

[CR42] Shin J, Lee J, Kim M-O (2014). Quantitative conductivity estimation error due to statistical noise in complex B + Map. JKSMRM.

[CR43] Shin J, Kim MJ, Lee J (2015). Initial study on in vivo conductivity mapping of breast cancer using MRI. J Magn Reson Imaging.

[CR44] Surowiec AJ, Stuchly SS, Barr JR, Swarup A (1988). Dielectric properties of breast carcinoma and the surrounding tissues. IEEE Trans Biomed Eng.

[CR45] Tha KK, Katscher U, Yamaguchi S (2018). Noninvasive electrical conductivity measurement by MRI: a test of its validity and the electrical conductivity characteristics of glioma. Eur Radiol.

[CR46] van Lier ALHMW, Hoogduin JM, Polders DL, et al (2011) Electrical conductivity imaging of brain tumours. In: Proceedings of the 19th annual meet ISMRM Montréal, Québec, Canada, p 4464

[CR47] Voigt T, Doessel O, Katscher U (2009) Imaging conductivity and local SAR of the human brain. In: Proceedings of 17th science international society for magnetic resonance in medicine Honolulu, Hawaii, USA, p 4513

[CR48] Voigt T, Katscher U, Doessel O (2011). Quantitative conductivity and permittivity imaging of the human brain using electric properties tomography. Magn Reson Med.

[CR49] Wen H (2003) Noninvasive quantitative mapping of conductivity and dielectric distributions using RF wave propagation effects in high field MRI. In: Proceedings of the SPIE 5030, medical imaging: physics of medical imaging. International Society for Optics and Photonics, pp 471–477

[CR50] Woo EJ, Seo JK (2008). Magnetic resonance electrical impedance tomography (MREIT) for high-resolution conductivity imaging. Physiol Meas.

[CR51] Yarnykh VL (2007). Actual flip-angle imaging in the pulsed steady state: a method for rapid three-dimensional mapping of the transmitted radiofrequency field. Magn Reson Med.

[CR52] Zhang X, Schmitter S, de Moortele VP (2013). From complex B1 mapping to local SAR estimation for human brain MR imaging using multi-channel transceiver coil at 7T. IEEE Trans Med Imaging.

[CR53] Zhang X, De Moortele PF, Van SS, He B (2013). Complex B1 mapping and electrical properties imaging of the human brain using a 16-channel transceiver coil at 7T. Magn Reson Med.

