# Supplementary material for: Brain Tissue Conductivity Measurements with MR-Electrical Properties Tomography: An In Vivo Study
Source: Brain Topogr. 2020 Dec 8;34(1):56–63. doi: 10.1007/s10548-020-00813-1 (PMC7803705; doi:10.1007/s10548-020-00813-1)
Supplement: Supplementary file 1 — Supplementary material 1 (PDF 1746 kb) [file 10548_2020_813_MOESM1_ESM.pdf]

SUPPLEMENTARY MATERIAL

Brain Tissue Conductivity Measurements with MR-Electrical Properties Tomography: An In Vivo Study

Stefano Mandija[1,2], Petar I. Petrov[3], Jord J.T. Vink[3], Sebastian F.W. Neggers[3], Cornelis A.T. van den Berg[1,2]

Affiliations

[1] Computational Imaging Group for MR diagnostic & therapy, Center for Image Sciences, University Medical Center Utrecht, Heidelberglaan 100, Utrecht, 3584 CX, The Netherlands

[2] Department of Radiotherapy, Division of Imaging & Oncology, University Medical Center Utrecht, Heidelberglaan 100, Utrecht, 3584 CX, The Netherlands

[3] Rudolf Magnus Institute of Neuroscience, University Medical Center Utrecht, Heidelberglaan 100, Utrecht, 3584 CX, The Netherlands.

Correspondence

S.Mandija@umcutrecht.nl

Journal:

Brain Topography

PART 1: IN SILICO HEAD EXPERIMENT

An in silico experiment was performed to characterize the impact of neglecting conductivity contributions arising from the computation of spatial derivatives through slices. Knowledge of the ground truth conductivity is available in silico. Therefore, correct assessment of the MR-EPT reconstruction accuracy could be performed.

METHODS

MR-EPT conductivity reconstructions were performed using the noise-robust 3D finite difference kernel K_{Large}^{3D}. This K_{Large}^{3D} kernel (7×7×5 voxels) is a combination of the in-plane "K_{Large}" used in the manuscript for in-plane derivatives (x/y-directions), and "K_{Small}" for derivatives through planes (z-direction), which are described in (van Lier ALHMW et al. 2012). By using a 3D kernel, the conductivity contribution arising from the computation of spatial derivatives through slices ($\partial^2 B_1^+ / \partial z^2$) was therefore included, contrary to the 2D in-plane kernel K_{Large} employed for the in vivo reconstructions from MRI measurements.

These reconstructions were performed for both noiseless data and data corrupted by noise (SNR = 50) (Figure SI1 below).

First, WM and GM mean conductivity values were computed after in-plane erosion by one voxel of the WM and GM masks. This erosion was performed to mimic the exclusion of the voxels at the interfaces between different tissue types (i.e. affected by partial volume effects).

Then, as for the in vivo cases in the manuscript, WM and GM mean conductivity values were recomputed after performing the same additional in-plane erosion of the WM and GM masks to discard regions affected by the in-plane boundary errors. The effect of this erosion on the WM/GM masks can be seen in the supplementary material part 3, Figure SI3 .

These results were ultimately compared to the WM and GM mean conductivity values reconstructed using the 2D in-plane kernel K_{Large}, thus allowing evaluation of the impact of using a 2D kernel instead of a 3D kernel on mean conductivity values.

RESULTS

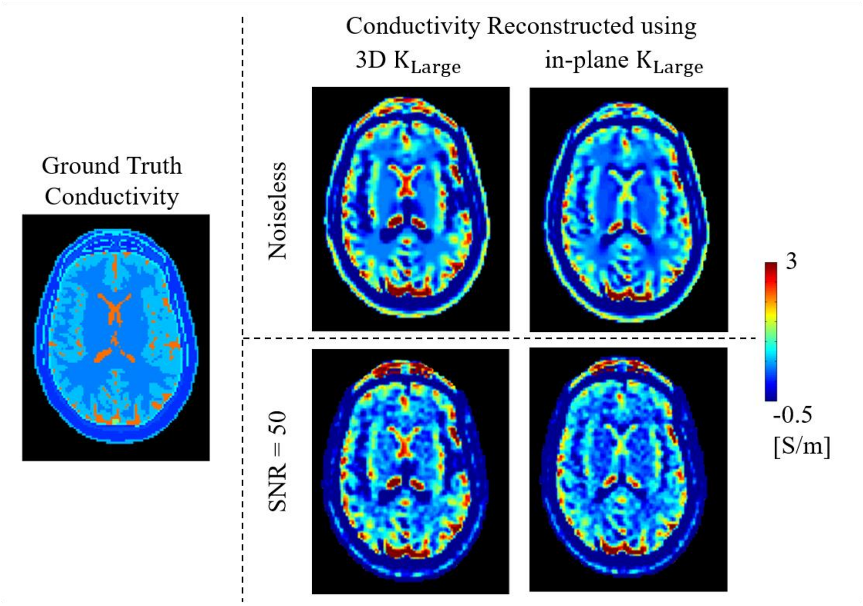

Figure SI1: Conductivity maps (example on one slice): ground truth (left) and reconstructed conductivity maps using respectively the 3D kernel (middle) and the in-plane kernel (right) K_{Large}. Notable the lower conductivity values, e.g. in the ventricles, when the in-plane kernel is adopted. Also notable the smaller spatial extension of boundary errors, e.g. undershooting around the ventricles (dark blue area), since boundary errors arising from spatial derivatives through slices are avoided when the in-plane kernel is adopted.

In figure SI1, examples of conductivity maps reconstructed using the 3D and the in-plane kernels K_{Large} are shown for the noiseless case and in presence of noise. From these maps, we can see the impact of noise in conductivity reconstructions (bottom row), and an overall underestimation when the 2D in-plane kernel is used (right column, see e.g. the ventricles). We can also notice the smaller spatial extension of boundary errors when the 2D in-plane kernel is used, see e.g. the undershooting area around the ventricles (dark blue), since boundary errors due to spatial derivatives through plane are avoided.

Conductivity reconstructions using 3D K_{Large}

After the first in-plane erosion, the reconstructed σ_{WM} and σ_{GM} mean ± standard deviation values are respectively 0.41±0.75 S/m and 1.31±1.90 S/m for the noiseless case, and respectively 0.42±0.78 S/m and 1.29±1.91 S/m in presence of noise. The observed overestimation is mainly due to the severe numerical boundary error introduced by the use of the large derivative 3D kernel K_{Large}^{3D}.

The overestimation of the mean values and the high standard deviations are reduced if the additional in-plane erosion is performed. The σ_{WM} and σ_{GM} mean ± standard deviation values after additional in-plane erosion are respectively 0.33±0.36 S/m and 0.77±0.86 S/m for the noiseless case, and 0.33±0.43 S/m and 0.78±0.88 S/m in presence of noise.

For the WM tissue, the good agreement between the mean σ_{WM} and the ground truth value (0.34 S/m) indicates that this in-plane erosion might be sufficient to minimize the impact of in-plane numerical boundary errors. Yet, boundary errors due to derivatives through slices are not fully excluded by in-plane erosion. These are the main cause of overestimation and large standard deviation for the GM tissue. The GM tissue is highly spatially convoluted. This has as a consequence that the computation of second order derivatives through slices ($\partial^2 B_1^+ / \partial z^2$) using the 3D kernel K_{Large}^{3D} leads to severe boundary errors, which are not fully excluded by the adopted 2D in-plane erosion.

This indicates that 3D Helmholtz EPT reconstructions are not suitable for small, highly spatially convoluted tissue structures like GM/CSF due to the large spatial extension of in-plane and through plane boundary errors (note that in-plane erosion does not resolve through plane boundary errors).

Comparison with the 2D in-plane K_{Large}

If conductivity maps are then reconstructed using the 2D in-plane K_{Large} employed in the manuscript for in vivo reconstructions after the same in-plane erosion is performed, the mean σ_{WM} and σ_{GM} ± standard deviation values are respectively 0.28±0.19 S/m and 0.51±0.34 S/m, noiseless case, and respectively 0.29±0.25 S/m and 0.52±0.42 S/m, in presence of noise.

This shows that the use of the 2D kernel K_{Large} leads to a small (~10%) underestimation in the reconstructed mean conductivity values with respect to the mean WM conductivity value reconstructed with the 3D Kernel (0.33 S/m), and with respect to the ground truth WM and GM conductivity values. This is attributable to the fact that the conductivity contribution arising from $\partial^2 B_1^+ / \partial z^2$ (~10%) is not considered when in-plane kernels are used. This results in an underestimation of the mean value for the benefit of avoiding severe numerical boundary errors through slices. In fact, provided the same in-plane erosion, the standard deviations of the 2D noiseless reconstructions are much lower compared to 3D noiseless reconstructions. This gives confidence that 2D reconstructions plus boundary erosion is suitable, provided sufficient area after erosion.

Conclusions

These results indicate that the performed in-plane erosion is sufficient to avoid in-plane boundary errors when the 2D in-plane K_{Large} is used, a the cost of a small (~10%) underestimation in the mean values, since the conductivity contribution arising from second order derivatives through slices is not considered (for more details see supplementary material part 2). This is particularly relevant for the GM region where, otherwise, second order derivatives through slices would lead to severe boundary errors (high overestimation of the mean σ_{GM} value).

PART 2: IN SILICO PHANTOM EXPERIMENT

To evaluate whether the position of the slice where conductivity maps are reconstructed with respect to the coil has an impact on the reconstructed conductivity values, 2D and 3D conductivity reconstructions were computed from noiseless in silico data obtained using a cylindrical phantom. Again, knowledge of the ground truth conductivity is available in silico, and therefore, correct assessment of the MR-EPT reconstruction accuracy could be performed.

The cylindrical phantom used for this simulation includes different cylinders with homogeneous conductivity. The conductivity values of the phantom are 0.1 S/m for background, while the inner cylinders have a conductivity value of 1, 2, 4 S/m, respectively. The middle slice of the phantom was placed in the center of the coil and the phantom was oriented along the z axis. By using a cylindrical geometry, boundary errors arising from through plane derivatives are avoided, thus allowing direct comparison between 2D and 3D reconstructions.

If the 3D kernel is used for conductivity reconstructions (first column), the reconstructed conductivity values match the ground truth independently from the chosen slice (absolute error in the second column is negligible, less than 1% after in-plane boundary erosion).

If the 2D in-plane kernel is used (third column), the same in-plane conductivity is also reconstructed for each slice, similarly to the previous case. However, the reconstructed conductivity maps show an underestimation with respect to the ground truth (see absolute errors, column four), since the contribution arising from spatial derivatives through slices is missing, regardless the slice being at the center or periphery of the cylinder.

The conductivity contribution due to derivatives through slices is shown in the fifth column. This is also the same, regardless the position of the slice being at the center or periphery of the phantom. This conductivity contribution is in the order of 10% for conductivity ranging between 0.1 and 2 S/m (human brain conductivity values).

This shows that the position of the slice where 2D conductivity reconstructions are performed does not impact the reconstructed values. Additionally, similarly to the head results, an underestimation (about 10%) is observed when the 2D kernel is used instead of the 3D kernel.

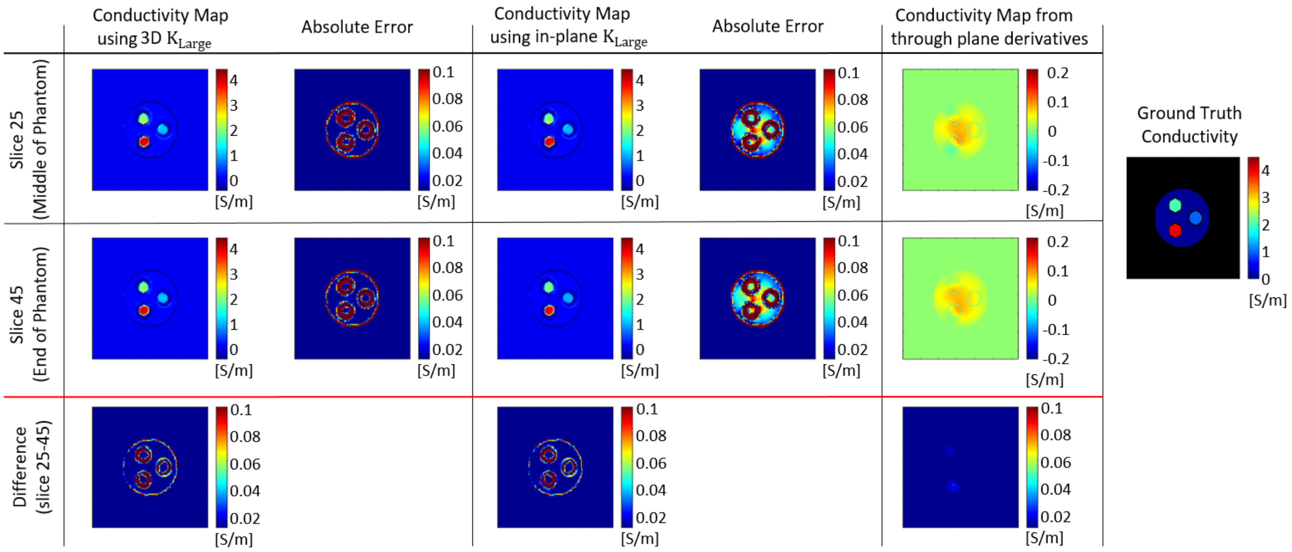

Figure SI2: Impact of slice position on 3D and 2D conductivity reconstructions (columns 1 and 3), and conductivity contributions arising from through plane derivatives (column 5). The difference maps between conductivity reconstructions on two different slices show that the impact of slice position on the reconstructed conductivity is negligible (last row). From the absolute error maps (columns 2 and 4), an underestimation of about 10% is observed when the 2D kernel is used instead of the 3D kernel. This underestimation corresponds to the conductivity contribution arising from derivatives through plane (column 5).

PART 3: IMPACT OF THE PERFORMED EROSION

The impact of the performed erosion is shown in the figure below for two slices: *left:* one slice in the middle of the Duke brain model, and *right:* one slice at the top of the Duke brain model.

For each slice, the impact of this erosion is shown for the WM (*top*) and the GM (*bottom*). First the initial masks are shown (*'initial' mask*), then the masks after erosion are shown (*'eroded' mask*). These eroded masks exclude regions at tissue boundaries affected by typical Helmholtz EPT reconstructions. Ultimately, the masks including the voxels removed by this erosion are shown ('*difference' mask*).

For the details on the functions adopted to perform this erosion (imerode and strel), we refer the reader to the description reported in the Matlab library.

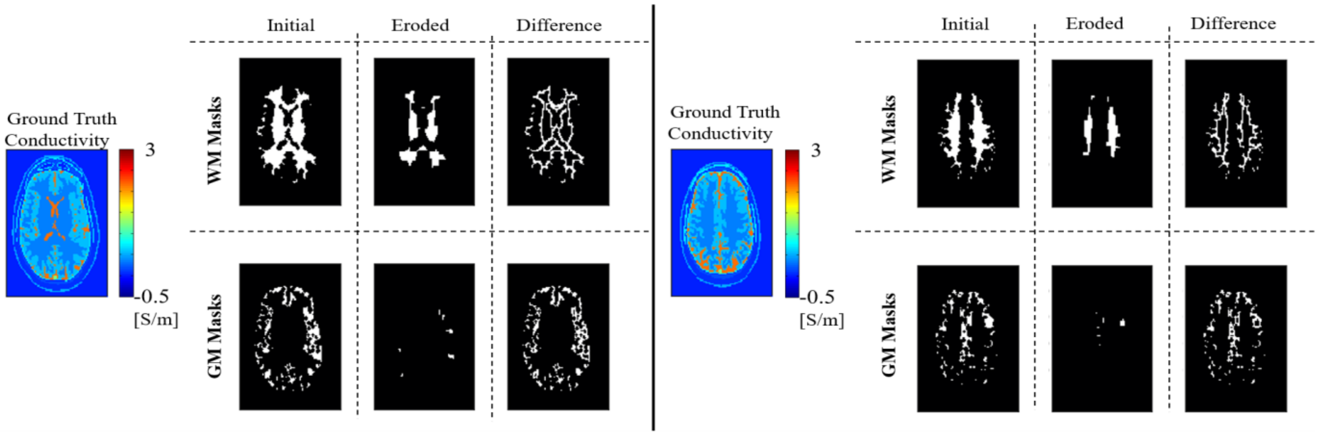

Figure SI3: Impact of the performed erosion on the WM and GM masks of the Duke brain model.

PART 4: ROI ANALYSIS FOR THE SAME BRAIN SLICE OF DIFFERENT SUBJECTS

For each subject, 9 regions of interest (ROIs) were taken in the slice used to show the conductivity reconstructions in figure 2 in the main manuscript. These ROIs were taken from the eroded tissue masks, and were overlaid on the Spin Echo T1-weighted images used as a reference. Mean and standard deviation values are reported for each ROI. For the WM, the ROIs (first 6 ROIs) were taken on similar regions across subjects. For the GM, this was not always possible. These values are in line with the reported values in table 1 (right side, i.e. after boundary erosion) in the manuscript.

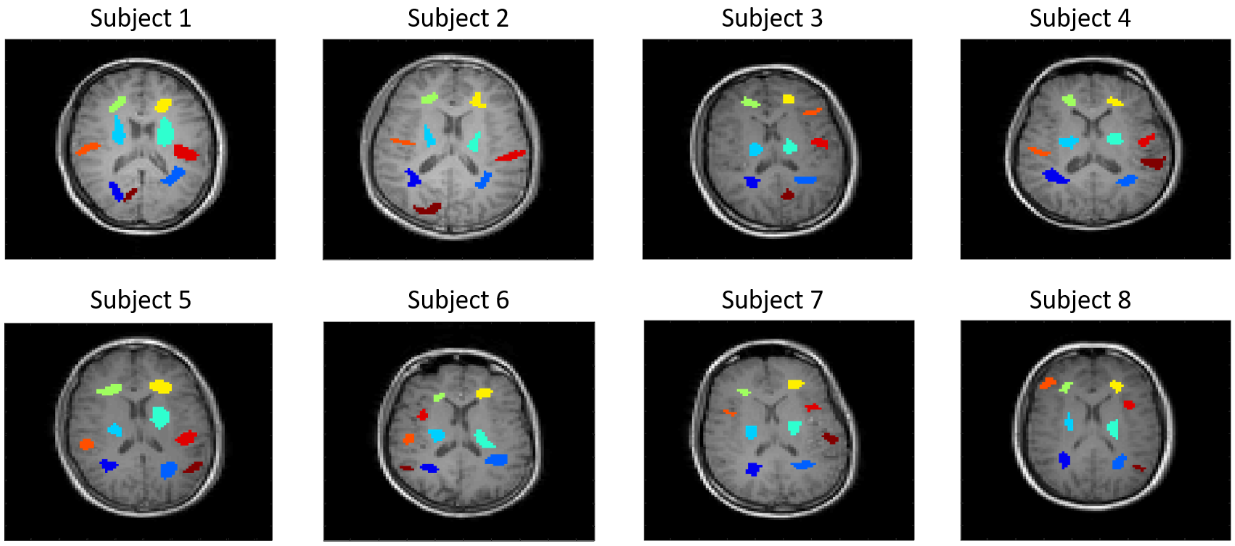

	Subject 1 [S/m]	Subject 2 [S/m]	Subject 3 [S/m]	Subject 4 [S/m]	Subject 5 [S/m]	Subject 6 [S/m]	Subject 7 [S/m]	Subject 8 [S/m]
ROI 1	0.28±0.36	0.34±0.32	0.29±0.24	0.29±0.14	0.33±0.14	0.34±0.09	0.32±0.25	0.32±0.07
ROI 2	0.35±0.43	0.33±0.20	0.30±0.27	0.29±0.10	0.32±0.18	0.30±0.19	0.31±0.09	0.34±0.11
ROI 3	0.35±0.11	0.30±0.10	0.30±0.14	0.30±0.19	0.30±0.07	0.33±0.26	0.34±0.18	0.32±0.18
ROI 4	0.30±0.17	0.28±0.11	0.32±0.17	0.28±0.19	0.28±0.15	0.28±0.14	0.29±0.17	0.30±0.12
ROI 5	0.31±0.08	0.29±0.12	0.33±0.37	0.34±0.12	0.30±0.27	0.29±0.17	0.30±0.06	0.31±0.21
ROI 6	0.30±0.16	0.28±0.11	0.30±0.28	0.30±0.16	0.34±0.23	0.30±0.31	0.30±0.09	0.33±0.25
ROI 7	0.54±0.75	0.50±0.27	0.50±0.24	0.47±0.21	0.54±0.47	0.55±0.23	0.51±0.22	0.48±0.55
ROI 8	0.50±0.78	0.49±0.26	0.56±0.93	0.50±0.48	0.55±0.51	0.49±0.61	0.53±0.36	0.48±0.41
ROI 9	0.52±0.20	0.50±0.19	0.48±0.20	0.46±0.82	0.48±0.26	0.50±0.33	0.54±0.53	0.46±0.28

Figure SI4: ROIs overlaid on normalized T1-weighted images [a.u.] as a reference, and mean ± standard deviation values [S/m] for each ROI of each subject. The adopted slices (top part) are the same as the ones used to show conductivity reconstructions for different subjects in figure 2 in the main manuscript.

PART 5: ROI ANALYSIS FOR DIFFERENT BRAIN SLICES OF SUBJECT 4

For subject 4, 3 regions of interest (ROIs) were drawn on different slices throughout the brain (the first 2 ROIs on the WM and the last ROI on the GM). As for part 4, these ROIs were taken from the eroded tissue masks, and were overlaid on the Spin Echo T1-weighted images used as a reference. Mean and standard deviation values are reported for each ROI. These values are in line with the reported values in table 1 (right side) in the main manuscript, after in-plane boundary erosion.

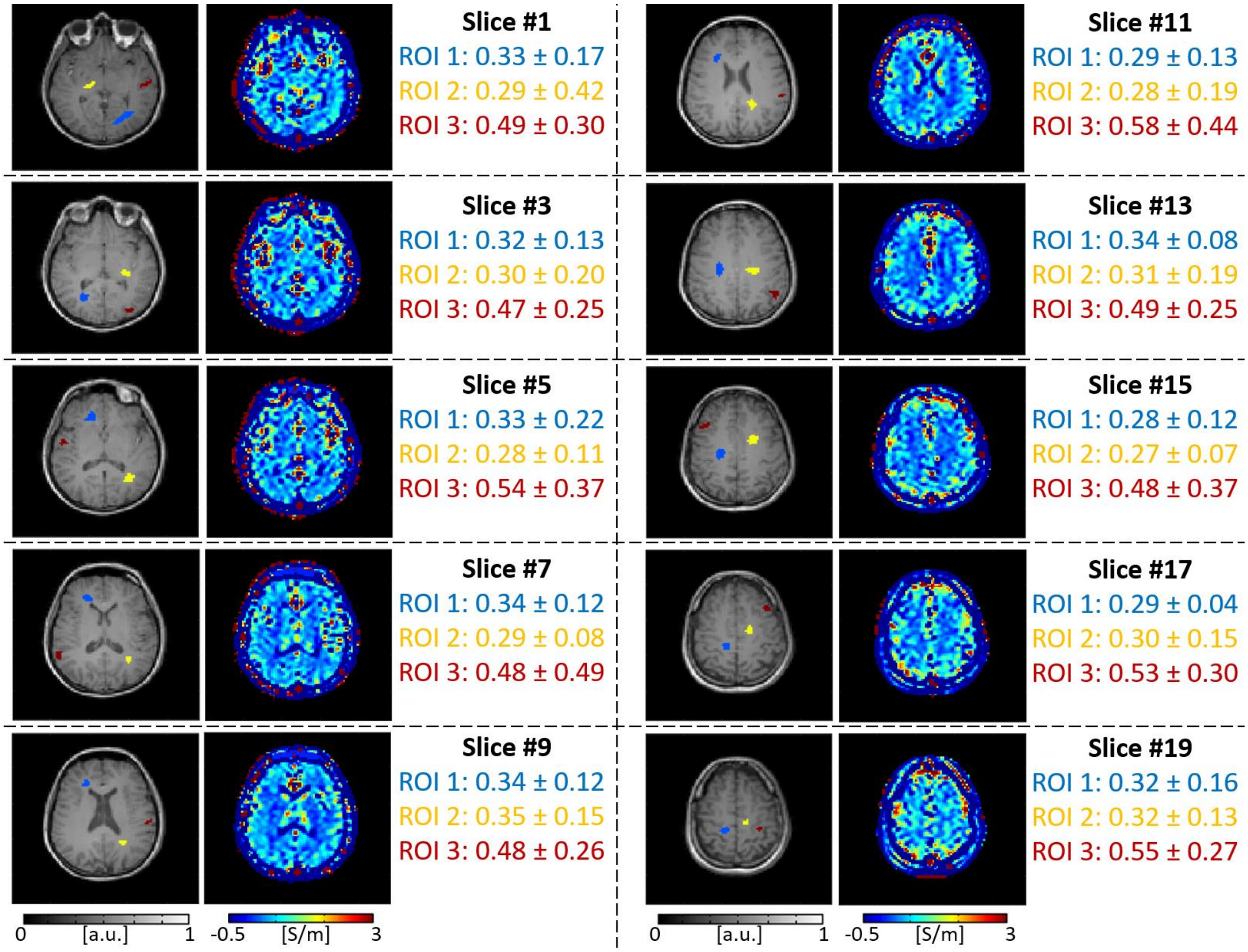

Figure SI5: ROIs overlaid on normalized T1-weighted images [a.u.] as a reference, and mean ± standard deviation values [S/m] for each ROI. These slices are taken throughout the brain of subject 4 as illustrative example.

REFERENCE

van Lier ALHMW, Brunner DO, Pruessmann KP, Klomp DWJ, Luijten PR, Lagendijk JJW, Van Den Berg CAT (2012) B1+ phase mapping at 7 T and its application for in vivo electrical conductivity mapping. Magn Reson Med 67:552–561. https://doi.org/10.1002/mrm.22995
